# Multiplex detection of ctDNA mutations in plasma of colorectal cancer patients by PCR/SERS assay

**DOI:** 10.7150/ntno.48905

**Published:** 2020-08-25

**Authors:** Nana Lyu, Vinoth Kumar Rajendran, Russell J. Diefenbach, Kellie Charles, Stephen J. Clarke, Alexander Engel, Sydney 1000 Colorectal Cancer Study Investigators*, Helen Rizos, Mark P. Molloy, Yuling Wang

**Affiliations:** 1 Department of Molecular Sciences, Macquarie University, Sydney, Australia.; 2 Department of Biomedical Sciences, Faculty of Medicine, Health and Human Sciences, Macquarie University, Sydney, Australia.; 3 Melanoma Institute Australia, Sydney, Australia.; 4 School of Medical Sciences, Discipline of Pharmacology, The University of Sydney, Australia.; 5 Royal North Shore Hospital, Department of Medical Oncology, The University of Sydney, Australia.; 6 Royal North Shore Hospital, Colorectal Surgical Unit, The University of Sydney, Australia.; 7 Bowel Cancer and Biomarker Laboratory, Kolling Institute, The University of Sydney, Australia.

**Keywords:** ctDNA, CRC, PCR/SERS assay, ddPCR, multiplex detection

## Abstract

Molecular diagnostic testing of KRAS and BRAF mutations has become critical in the management of colorectal cancer (CRC) patients. Some progress has been made in liquid biopsy detection of mutations in circulating tumor DNA (ctDNA), which is a fraction of circulating cell-free DNA (cfDNA), but slow analysis for DNA sequencing methods has limited rapid diagnostics. Other methods such as quantitative PCR and more recently, droplet digital PCR (ddPCR), have limitations in multiplexed capacity and the need for expensive specialized equipment. Hence, a robust, rapid and facile strategy is needed for detecting multiple ctDNA mutations to improve the management of CRC patients. To address this significant problem, herein, we propose a new application of multiplex PCR/SERS (surface-enhanced Raman scattering) assay for the detection of ctDNA in CRC, in a fast and non-invasive manner to diagnose and stratify patients for effective treatment.

**Methods:** To discriminate ctDNA mutations from wild-type cfDNA, allele-specific primers were designed for the amplification of three clinically important DNA point mutations in CRC including KRAS G12V, KRAS G13D and BRAF V600E. Surface-enhanced Raman scattering (SERS) nanotags were labelled with a short and specific sequence of oligonucleotide, which can hybridize with the corresponding PCR amplicons. The PCR/SERS assay was implemented by firstly amplifying the multiple mutations, followed by binding with multicolor SERS nanotags specific to each mutation, and subsequent enrichment with magnetic beads. The mutation status was evaluated using a portable Raman spectrometer where the fingerprint spectral peaks of the corresponding SERS nanotags indicate the presence of the mutant targets. The method was then applied to detect ctDNA from CRC patients under a blinded test, the results were further validated by ddPCR.

**Results:** The PCR/SERS strategy showed high specificity and sensitivity for genotyping CRC cell lines and plasma ctDNA, where as few as 0.1% mutant alleles could be detected from a background of abundant wild-type cfDNA. The blinded test using 9 samples from advanced CRC patients by PCR/SERS assay was validated with ddPCR and showed good consistency with pathology testing results.

**Conclusions:** With ddPCR-like sensitivity yet at the convenience of standard PCR, the proposed assay shows great potential in sensitive detection of multiple ctDNA mutations for clinical decision-making.

## Introduction

Colorectal cancer (CRC) is the third most commonly diagnosed malignancy (after breast cancer and prostate cancer) and the second leading cause of cancer deaths (after lung cancer) in Australia with 16,398 new cases and 5,597 deaths estimated in 2019 1. The high incidence develops through a multistage process where a series of cellular mutations occur over time. Epidermal growth factor receptor (EGFR) signalling pathway plays an important role in the development and progression of CRC. The clinical outcome of patients with metastatic CRC has been improved by introduction of two anti-EGFR targeted monoclonal antibodies, cetuximab and panitumumab 2-4. However, the overexpression of EGFR and subsequent hyperactivation of mitogen‑activated protein kinase (MAPK) signalling is insufficient to predict a therapeutic response when these antibodies were used clinically 3, 5. This is often due to the activating mutations of EGFR downstream signalling effectors, such as KRAS and BRAF, which are commonly activated oncogenes in CRC pathogenesis and are sufficient to cause persistent hyperactivation of MAPK proliferative pathway regardless of EGFR inhibition. Mutant tumors are associated with poor prognosis, lower response to standard chemotherapy and these patients derive no benefit from anti-EGFR treatment 6, 7. Therefore, clinical use of anti-EGFR inhibitors cetuximab and panitumumab requires prior determination of wild-type KRAS and BRAF genotype 8. Clinical mutation testing commonly uses resected tumor specimens for DNA sequencing of target genes or mutation hotspots or in some cases such as BRAF, immunohistochemistry (IHC) can be used 9. ctDNA based testing outside of clinical research has not yet been endorsed in cancer guidelines, although given the rapid developments from intense research 10.

Circulating cell-free DNA (cfDNA) is shed into the bloodstream from normal and tumor cells, and the fraction of cfDNA derived just from tumor cells is referred to as circulating cell-free tumor DNA (ctDNA). The use of ctDNA has come into focus as a source of non-invasive material to provide real-time information with respect to the assessment of minimal residual disease, treatment response, prognosis and resistance mechanisms 11, 12. ctDNA maintains the same genomic signatures that are present in the corresponding tumor tissue, analysis of ctDNA has the potential to change clinical practice by exploring blood rather than tissue, as a source of diagnostic information 13. There has been intense research of ctDNA in CRC for numerous diagnostic, predictive, and prognostic applications in recent years 14-16. Currently, the most common methods used to detect ctDNA mutations include real-time PCR, digital PCR, and next-generation sequencing (NGS). While highly accurate, these fluorescence-based or NGS-based approaches require long analysis time, and expensive specialized equipment and long operation time (3-4 hours) 17-20. Thus, alternative approaches to address the limitations of the current techniques are required.

Herein, we describe a new application of multiplex PCR/SERS assay based on allele-specific PCR and surface-enhanced Raman scattering (SERS) to address the limitations of fluorescence-based DNA detection for CRC. SERS is a vibrational spectroscopic technique for probing molecules on or near the nanoscale surface of metallic substrates, which enables a rapid, sensitive and non-destructive detection of the target molecules with characteristic spectra via localized surface plasmon resonances 21-24. Recently, SERS nanotags as a new class of label have demonstrated unique and attractive applications in biological labelling with key advantages including ultra-sensitivity (down to single molecule under certain conditions), suitability for multiplexing due to the narrow width of vibrational Raman bands, quantification based on spectral intensities, high photostability, minimal autofluorescence from biological specimens via red to near-infrared (NIR) laser excitation, and the requirement for only single laser source for excitation 21, 22, 25-27. The multiplexing capability is potentially very useful for screening multiple mutations from limited samples, such as ctDNA. In addition, the need for only single laser excitation endows its great potential to be used in real patient cases due to simpler and low-cost instrumentation. Most SERS applications for identifying mutations are based on the combination of SERS-active particles with elaborately designed molecular beacon probes which turn “on” or “off” the SERS signal 28-30. The fluorescence emission of the molecular beacons tends to be overlapping due to the broad emission profile of fluorophores, resulting in the difficulty in deconvolution of mixed signals, which limits the multiplexing capability 31. On the other hand, most PCR-based SERS applications use labelled DNA probes to hybridize with the PCR amplicons during annealing process, followed by tedious processes to remove unincorporated probes and other impurities 32.

In this study, we proposed the new application of a simple multiplex PCR/SERS assay for detection of ctDNA mutations in CRC. PCR/SERS assay was constructed based on allele-specific PCR for amplification of common CRC mutant targets including KRAS G12V (c.35G>T), KRAS G13D (c.38G>A) and BRAF V600E (c.1799T>A), followed by SERS nanotags identification of the mutant targets and SERS signal readout of the corresponding mutations. The PCR/SERS assay was first validated by genotyping CRC cell lines and plasma-derived cfDNA. The detection specificity and sensitivity were also assessed, where the specific amplification of targets was observed in PCR-based gel electrophoresis and the sensitivity of PCR/SERS assay was demonstrated as low as 0.1% mutant allele frequency (MAF) for plasma cfDNA. The detection of cfDNA from CRC patients were performed under blinded test conditions, the results from our assay were further validated by droplet digital PCR (ddPCR). Our assay showed consistent results with ddPCR, but with the added convenience of single tube multiplex assay and reduced time compared to ddPCR.

## Results and Discussion

### Multiplex PCR/SERS assay

**Figure 1:**
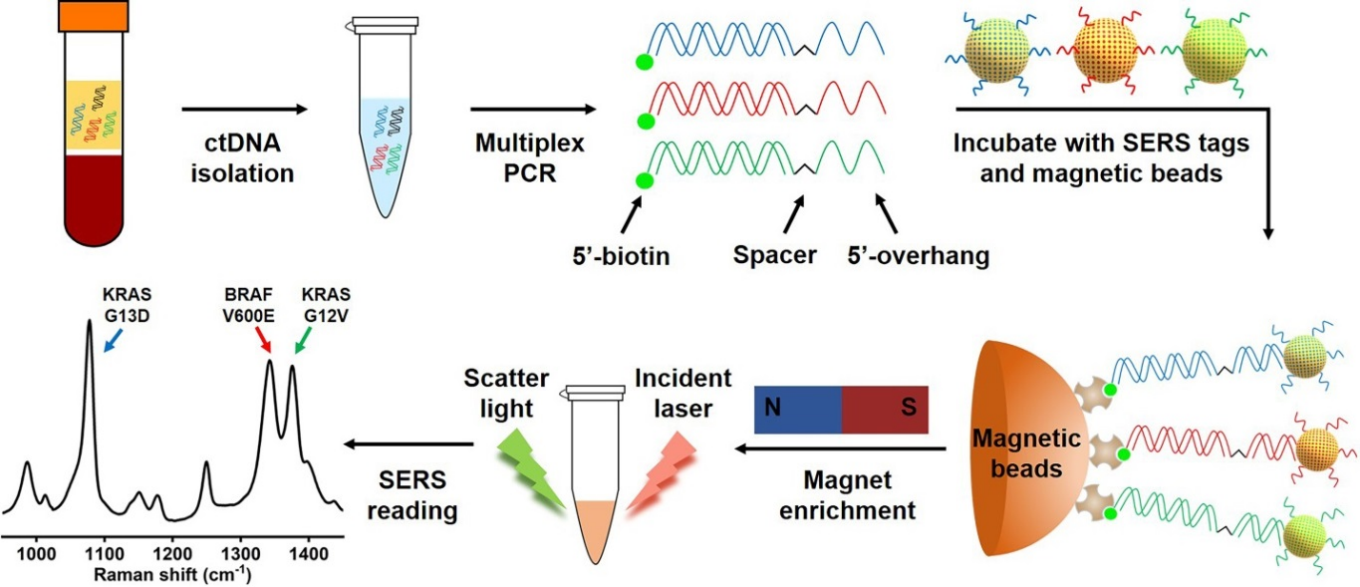
** Scheme of the multiplex PCR/SERS assay.** Multiplex mutation-specific primers were used to amplify mutant targets (the wild-type dsDNA is shown in black). Amplicons were then labelled with mutation-specific nanotags and enriched with streptavidin magnetic beads. The status of mutations was then analysed with SERS spectrum where unique spectral peaks demonstrated the presence of targeting mutations.

Mutations in the KRAS oncogene are found in 35%-45% of CRC, and mutations of BRAF are present in ~10% of CRC cases 3, 33. In addition to their critical role in driving tumorigenesis, these mutations have been demonstrated to be strong negative predictors for response of CRC to anti-EGFR therapy 3, 7, 33, 34. Thus, three clinically important DNA point mutations in CRC, KRAS G12V (c.35G>T), KRAS G13D (c.38G>A) and BRAF V600E (c.1799T>A), were selected as targets to test our method. We designed allele-specific primers with a single nucleotide polymorphism (SNP) at the 3'-end of the primers to distinguish mutant targets from the wild-type background, where only mutant targets were amplified by the polymerase (**Table S1**). In each pair of primers, one primer had a 15-oligonucleotide unique sequence followed by an internal carbon spacer upstream of the allele-specific sequence. 5'-overhang after PCR was effectively created as DNA polymerase cannot extend beyond the carbon spacer on the reverse strand 18. The second PCR primer is modified with 5'-biotin which allows binding to streptavidin coated magnetic beads (SMB), leading to the enrichment of amplicons by the beads (**Figure 1**). In the presence of the target mutation, effective PCR amplification will occur, and the resulting amplicon will have a biotin handle on one end and a 5'-overhang on the other end. SERS nanotags are gold nanoparticles (AuNPs) coated with Raman molecules and have attached a DNA probe which is complementary to the unique 5'-overhang oligonucleotide barcode sequence of each amplicon (**Table S1**) to form a SERS nanotag/amplicon/biotin complex. The excessive SERS nanotags are then removed after enrichment of the targeting amplicons with streptavidin magnetic beads, the presence of targeting amplicons is then ascertained with a portable Raman spectrometer, where the fingerprinting spectrum indicates the presence of the SERS nanotags, which reflects the presence of the targeting mutation (**Figure 1**). Raman molecules used in this study were 2,3,5,6-tetrafluoro-4-mercaptobenzoic acid (TFMBA) for KRAS G12V at Raman shift of 1376 cm^-1^, 4-mercaptobenzoic acid (MBA) for KRAS G13D at Raman shift of 1076 cm^-1^, and 5,5'-dithiobis-(2-nitrobenzoic acid) (DTNB) for BRAF V600E at Raman shift of 1342 cm^-1^. The small size of aromatic molecules (TFMBA, MBA and DTNB) with thiols group were selected because of their ability to conjugate directly to gold surfaces via stable Au-S bonds, which lead to the formation of self-assembled monolayers (SAMs) on gold surfaces. Reproducible SERS signatures are obtained due to the dense packing and uniform orientation of the Raman reporter molecules on AuNPs surface. Additionally, the multiplex detection could be achieved owing to the appearance of only a few distinct vibrational Raman peaks 21, 35. The most intensive Raman peaks at 1376 cm^-1^ for TFMBA, 1076 cm^-1^ for MBA and 1342 cm^-1^ for DTNB ensure SERS labels be easily distinguished.

### Specificity study

**Figure 2:**
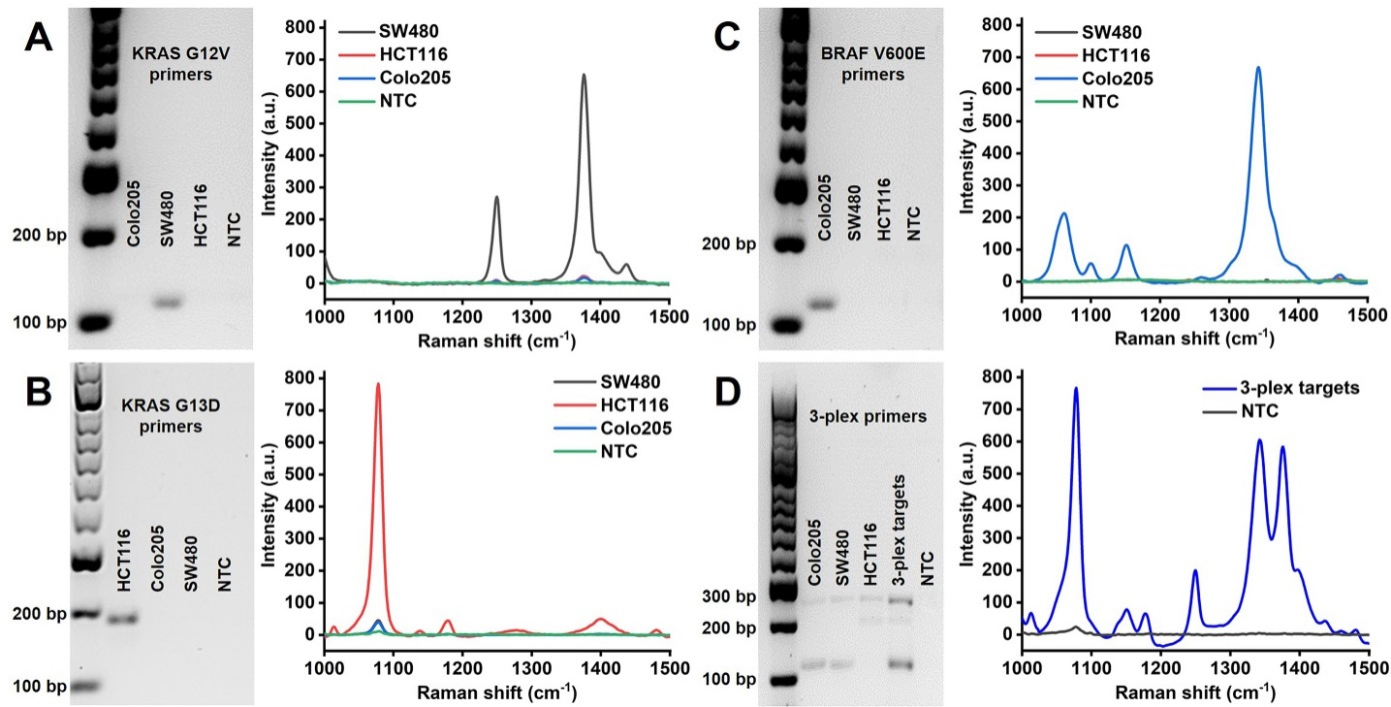
** Specific amplification of the mutant targets by PCR and specific identification of PCR amplicons by SERS nanotags.** (A, B, C) Left: Gel electrophoresis verified the specific amplification of the mutant gDNA with the corresponding individual primers, where no amplification of the wild-type gDNA was observed. Right: Specific detection of individual mutant PCR products with the corresponding single SERS nanotags; (D) Multiplex detection of the mutant targets with 3-plex primers and 3-plex SERS nanotags. NTC is the no template control.

Amplification specificity is crucial for DNA mutations with single base changes. The proposed PCR/SERS assay was based on the allele-specific PCR, followed by identification with probe labelled SERS nanotags. In this study, we developed a 3-plex PCR assay for KRAS G12V, KRAS G13D and BRAF V600E mutations. Specific primers (**Table S1**) targeting each mutation were designed with minor modifications (change of primer length, or design of different forward/reverse primers to optimize specificity) based on published reports 18, 36, 37. The specificity of each pair of primers was confirmed using genomic DNA (gDNA) from SW480 (KRAS G12V), HCT116 (KRAS G13D) and Colo205 (BRAF V600E) cell lines. As indicated by the agarose gel electrophoresis, only the bands representing the target mutation were present in the gel image, the amplicon size for KRAS G12V, KRAS G13D and BRAF V600E are 92 bp, 177 bp and 100 bp, respectively. With the modification of overhang and biotin in the primers, the mobility of obtained amplicons increased, causing shift in the expected position (**Figure 2**). The results of gel electrophoresis demonstrate the excellent specificity of the primers for amplifying the corresponding mutant gDNA from cells. The corresponding SERS spectra of target mutations were observed as TFMBA at 1376 cm^-1^ for KRAS G12V, MBA at 1076 cm^-1^ for KRAS G13D and DTNB at 1342 cm^-1^ for BRAF V600E. The specific SERS profile indicated the effective identification of each SERS nanotag for the corresponding mutation, thus demonstrating the specificity of PCR/SERS assay for the detection of specific mutations (**Figure 2**).

While previously considered mutually exclusive, concomitant mutations in both KRAS and BRAF genes have been reported in CRC, which may have profound clinical implications for disease progression and therapeutic responses 38, 39. Thus, to further demonstrate the specificity of the assay for multi-mutant tumor, PCR assay with 3-plex primers for a mixture of all three mutant targets was performed, followed by SERS assay with 3-plex mixture of SERS nanotags (**Figure 2D**). The three distinct SERS peaks from the 3-plex nanotags were observed, representing the presence of the three input targets, while no signal was detected for the no template control (NTC).

The specificity of multiplex (duplex and triplex) PCR amplification was also validated with gel electrophoresis (**Figure S1**), in which the presence of target amplicons was observed after multiplex PCR, which could genotype cell lines accurately. It was noted that the duplex amplification with KRAS 12V and KRAS G13D and 3-plex amplification showed an additional band at approximately 300 bp (**Figure S1**), which may be due to cross reaction of primers for the targets, where the KRAS G12V forward primer and KRAS G13D reverse primer amplified the template and obtained an additional amplicon with length of 229 bp. To avoid this additional amplicon being identified with SERS nanotags, the KRAS G12V forward primer and KRAS G13D reverse primer were both designed with a 5'-biotin modification (**Figure S2**). Consequently, the additional amplicon had 5'-biotin on both ends but without 5'-overhang oligonucleotides would not allow binding to the SERS nanotags in the following SERS assay. For KRAS G12V (c.35G>T) and KRAS G13D (c.38G>A), the mutant position is very close on the DNA sequence, many primers were optimized to obtain specific identification for these two targets, and eventually we designed the specific primers described in this study (**Table S1**).

### Sensitivity study

**Figure 3:**
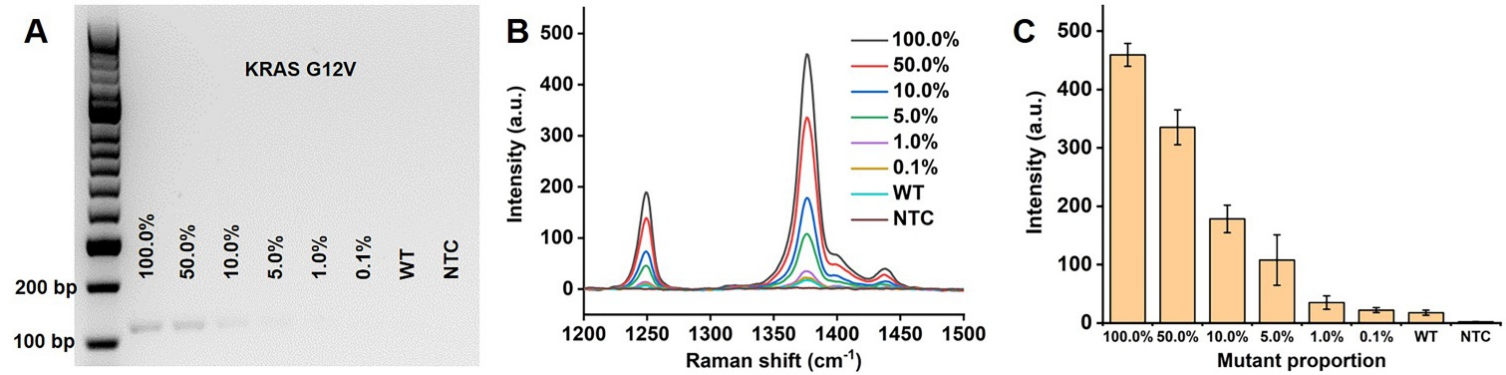
** Detection of low levels of KRAS G12V mutation load.** (A) Gel electrophoresis image, (B) typical raw Raman spectra and (C) bar graph of average SERS intensities at 1376 cm^-1^ over a range of mutation loads for 10,000 input copies. NTC is the no template control. Error bar represents standard deviation (SD) of 3 independent experiments.

High sensitivity methods are required for detection of early cancer with rare mutant targets from the background of normal DNA. To evaluate the sensitivity of our proposed PCR/SERS assay, known copies of the mutant sequences were spiked in the background wild-type templates (10,000 copies in total). To further increase the complexity of the system, gDNA extracted from cells was adopted as the wild-type templates. Taking KRAS G12V as an example, as few as 0.1% mutant sequences (10 copies) could be detected over the wild-type background (gDNA from Colo205, which is wild-type for KRAS G12V) and no template control (**Figure 3**). This level of sensitivity (0.1%) in detecting low input targets achieved by our method, was 50 times higher than that of the standard PCR-based agarose gel electrophoresis (5.0% mutant allele frequency, MAF, the ImageJ software was used for band quantification to estimate the cut-off value). The same sensitivity was also observed for KRAS G13D and BRAF V600E (**Figure S3, S4**). The high sensitivity of this assay could be attributed to the bright SERS nanotags used in our study, the enhancement factor of the AuNPs (60 nm) for Raman reporter TFMBA was estimated to be 5.49×10^6^ (**Calculation, Supplementary Material**) 22, 40-42. The negligible SERS signal from 100% wild-type templates further validate the specificity of primers for PCR amplification.

**Figure 4:**
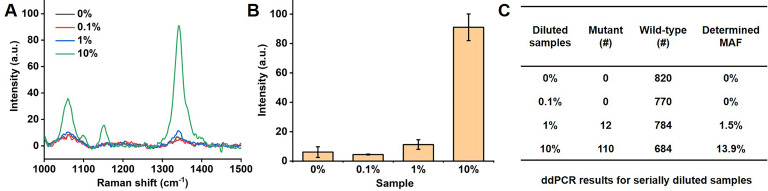
** Detection of serially diluted BRAF V600E mutation.** (A) Typical raw Raman spectra, (B) Bar graph of average SERS intensities at 1342 cm^-1^ over a range of 0% to 10% of BRAF mutant allele frequency (MAF). Error bar represents SD of 3 independent experiments. (C) ddPCR results for serially diluted samples (# copy number per sample, 4 ng of template was used in each case).

To further evaluate the sensitivity of our PCR/SERS assay, we performed the PCR/SERS assay and ddPCR on the same serial samples, as indicated in** Figure 4**. ctDNA from a BRAF V600E patient with a known MAF was serially diluted with cfDNA from a healthy donor (HD) to obtain samples with a MAF of 0% (HD), 0.1%, 1% and 10%, then analysed with PCR/SERS (2 ng/µL of cfDNA, add 2 µL for PCR) and compared with ddPCR. The results from PCR/SERS assay showed that the sample with 1% MAF could be distinguished from healthy cfDNA, while 0.1% MAF could not be distinguished, which may be due to the sample loss during the serial dilutions. The results were further validated by ddPCR (4 ng of cfDNA was used for each sample), thus demonstrating the accurate and sensitive detection of mutant targets in ctDNA by PCR/SERS assay (**Figure 4**).

### Detection of ctDNA mutations from colorectal cancer patients

**Figure 5:**
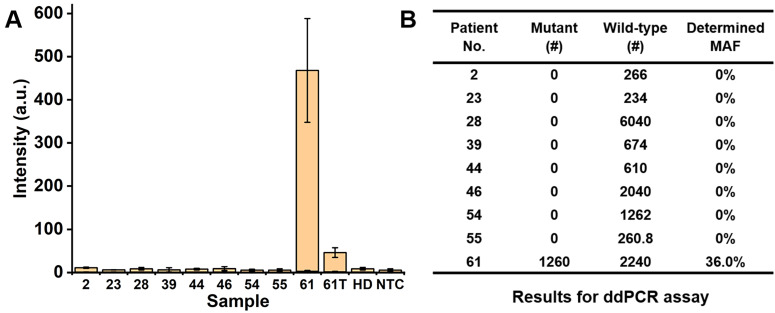
** Detection of BRAF V600E mutation using cfDNA from CRC patients.** (A) Bar graph of average SERS intensities at 1342 cm^-1^ for patients' samples, where 61T represents the patient #61 after 4 months of chemotherapy. (B) Results for ddPCR assay (# copy number per sample).

The PCR/SERS assay was further applied for detecting cfDNA mutations from 9 CRC patients (8 for Stage IV, 1 for Stage III), as shown in **Table 1**. In a blinded experiment, the PCR was performed with 3-plex primers (for KRAS G12V, KRAS G13D and BRAF V600E) and 4 µL of cfDNA (concentrations of cfDNA are listed in **Table 1**, used directly without dilution for PCR/SERS assay and ddPCR). Among the 9 patients' plasma, two Stage IV samples were detected with BRAF V600E mutation, with no evidence of KRAS G12V or KRAS G13D mutations found in any sample. The proposed PCR/SERS assay showed equivalent results with ddPCR detection (9.9 µL of cfDNA was used in each case) we used for validation of findings (**Figure 5, and Figure S5**). When we unblinded the results, strong PCR/SERS BRAF V600E signal for patient #61 was confirmed by IHC and sequencing. The seven remaining Stage IV cases were BRAF and KRAS wild-type which agreed with pathology reporting. The Stage III case (patient #46) was BRAF V600E positive by IHC but our test sample obtained at the 4^th^ chemotherapy cycle (5-fluorouracil based doublet chemotherapy) did not show evidence of BRAF V600E ctDNA, which may correlate with good prognosis for that patient. We also followed patient #61 during treatment and after 4 months of chemotherapy, we observed ten-fold reduced ctDNA levels for the BRAF mutant allele, consistent with good treatment response. It has been reported that serial monitoring of BRAF V600E levels in ctDNA at baseline and on treatment may be a clinically useful marker for tumor response, with greater reduction in ctDNA mutant alleles in responding patients compared to those with stable or progressive disease 43-45. These results demonstrate that the PCR/SERS ctDNA detection strategy holds great potential for rapid liquid biopsy mutation detection, tracking treatment responses, monitoring tumor progression, and assessing residual disease. It should also be pointed out that although PCR/SERS assay has shown the great advantages in the sensitivity, specificity and multiplexed capability for ctDNA detection, the key concern remains in detecting the multiple single point mutations where the two mutants are very close on the DNA sequence. For instance, to achieve the specific identification of the KRAS G12V (c.35G>T) and KRAS G13D (c.38G>A) point mutations which are very close on the DNA sequence, it is essential to design highly specific primers to avoid false positives.

## Conclusion

In summary, we have demonstrated a strategy using PCR/SERS for multiplex detection of clinically important CRC mutations from patient cfDNA. Compared to the multi-tube sample reactions which are required to evaluate multiple mutations, PCR/SERS assay described herein could interrogate at least 3 mutation targets per tube. We believe that this strategy could readily be exploited for higher multiplexing capability due to the benefits of SERS technique 10, 46, 47. The approach meets the criteria of being facile, sensitive (down to 0.1% MAF) and specific for multiplex detection of ctDNA mutations. Our results are consistent with findings from ddPCR, while PCR/SERS provides a simplified and cheaper workflow using a portable detector without the need of ddPCR instrumentation. Thus, we believe this PCR/SERS strategy is a competitive candidate for multiplex detection of ctDNA mutations in both research and clinical diagnostics.

## Material and Methods

### Cell line DNA sample preparation

CRC cell lines including SW480, Colo205 and HCT116 were purchased from American Type Culture Collection (ATCC). Genomic DNA (gDNA) was purified with DNeasy Blood and Tissue Kit (Qiagen) as instructed by the manufacturer. The concentration of gDNA was determined by spectrometry (NanoDrop, Thermo Scientific) and 50 ng were used as input for PCR/SERS assay.

### Patient plasma cfDNA samples

This study was conducted according to the National Health and Medical Research Council Australian Code for the Responsible Conduct of Research and the National Statement on Ethical Conduct in Human Research. All patients have provided their written informed consent to provide samples and linked data for research which was approved by Sydney Local Health District - CGRH Human Research Ethics Committee (CH62/6/2016-027). This subproject was approved by the Human Research Ethics Committee of Macquarie University (Project ID: 5392). The healthy blood was drawn from one healthy donor with ethics approval from Macquarie University Human Research Ethics Committee (Project ID: 0596). The blood (10 mL) was collected in EDTA tubes (Becton Dickinson) and processed within 4 h from blood draw. Tubes were spun at 800×g for 15 min at room temperature (RT). Plasma was then removed into new 15 mL tubes without disturbing the buffy coat and re-spun at 1600×g for 10 min at RT to remove cellular debris. Plasma was stored in 1-2 mL aliquots at -80°C, in which the ctDNA are stable for the following analysis.

For CRC patients and healthy donor, cfDNA was purified from 1 mL frozen aliquot of the plasma using the QIAamp Circulating Nucleic Acid Kit (Qiagen) as instructed by the manufacturer, 30 µL of Buffer AVE was used to elute cfDNA from the QIAamp Mini Column. cfDNA amounts were then determined by the Qubit dsDNA High Sensitivity Assay Kits (Thermo Scientific). Generally, 0.4-2 ng/µL of cfDNA was obtained, 2 or 4 µL was then used in the PCR/SERS assay.

### Preparation of SERS nanotags

SERS nanotags were prepared according to our previous report 48. Gold nanoparticles (AuNPs) with diameter of 60 nm were synthesized by citrate reduction of HAuCl_4_ 49. SERS nanotags were prepared by modifying AuNPs with Raman reporters and DNA oligonucleotides. Briefly, 1.5 mL AuNPs were concentrated into 1 mL and mixed with 10 μL Raman reporter ethanolic solution (1 mM) at RT for 2 h. Then, 50 μM TCEP activated thiolated DNA oligonucleotides (IDT) was added to the AuNPs and incubated at RT for overnight to obtain SERS nanotags. Then, 0.6 M NaCl in 1 mM PBS was used to age the SERS nanotags at RT for 12 h. After the salt aging step, stable oligonucleotide-modified SERS nanotags were obtained and could be stored at 4°C for several weeks. Finally, SERS nanotags were centrifuged and resuspended into 1 mM PBS solution prior to use on the SERS assay. The oligonucleotides are listed in **Table S1**. Successful functionalization of SERS nanotags were confirmed with UV-Vis spectrometry (**Figure S6**). The enhancement factor of AuNPs to the Raman molecules was estimated to be 10^6^-10^8^ 22, 40-42, detailed calculation was described in the **Supplementary Material**.

### PCR/SERS assay

Multiplex PCR was performed using the KAPA2G Robust HotStart PCR Kit (Kapa Biosystems) with minor modification according to our previous report 18. The allele-specific primers used in this study are listed in **Table S1**. Each 20 μL reaction contained 1.25× Buffer A, 3.125 mM MgCl_2_, 1.8 M ethylene glycol, 1.6 μg BSA, 75 nM or 50 nM of each primer, 0.2 mM of each dNTP and 0.4 U of polymerase (**Table S2**). Thermal cycling conditions were 94°C for 5 min followed by 31 cycles of 94°C for 30 s, 55°C for 30 s and 72°C for 30 s, and then a final extension at 72°C for 2 min. After PCR, 4 μL of products were loaded in 2% agarose gel containing GelRed Nucleic Acid Stain (4 μL stain per 100 mL gel) for electrophoresis in Tris-acetate-EDTA buffer to confirm PCR amplification. The remaining PCR products were used for SERS detection. Three independent experiments were performed for PCR/SERS assay.

3 μL of SERS nanotags mix was added to each 10 μL PCR sample and incubated at 300 rpm, 35°C for 15 min. Subsequently, 10 μL of streptavidin coated magnetic beads (SMB, S1420S, New England Biolabs) was added to the PCR/SERS mix and left to incubate for another 10 min at 300 rpm, RT. The PCR/SERS/SMB complex was then isolated with a magnet and washed 3 times by resuspending the pellet with 0.25× PBS supplemented with 0.01% Tween 20 (30 μL buffer for each wash). After the final wash, the pellet was then resuspended in 60 μL of 2× PBS and transferred to a quartz cuvette prior to SERS measurement on the IM-52 portable Raman microscope (Snowy Range Instruments). SERS spectra were obtained from five 4-second acquisitions using a 785 nm excitation laser at 70 mW.

### ddPCR analysis of cfDNA from plasma

The copy number of cfDNA was determined using the QX200 droplet digital PCR (ddPCR) (Bio-Rad) system to detect tumor-associated BRAF V600E mutation, as previously described 50. Briefly, the reaction mixture (supermix, primers/probe, cfDNA sample and water) were prepared according to the manufacturer's instruction. The QX200 Droplet Generator (Bio-Rad) partitions each 20 µL reaction mixture into more than 10,000 nanoliter-sized water-in-oil droplets for PCR amplification, which was performed using the following conditions: 1 cycle of 95°C for 10 min, 40 cycles of 94°C for 30 s and 55°C for 1 min, and 1 cycle of 98°C for 10 min. Following amplification, droplets from each sample were analysed individually on the QX200 Droplet Reader (Bio-Rad), where PCR-positive and PCR-negative droplets are counted to provide absolute quantification of target DNA in digital form. The DNA copy number per 20 µL reaction for the mutant and wild-type circulating DNA species was determined with Quantasoft software version 1.7.4 (Bio-Rad, Hercules, CA, USA) using a manual threshold setting. All samples with fewer than three positive mutant droplets were considered negative to improve specificity.

## Supplementary Material

Supplementary figures and tables.

## Figures and Tables

**Table 1:** Detection of BRAF V600E from colorectal cancer patients' cfDNA by PCR/SERS assay and ddPCR detection

Patient No.^a^	Stage	Tissue genotyping	cfDNA (ng/µL)	PCR/SERS assay	ddPCR MAF
2	IV	Wild-type	0.212	Negative	0%
23	IV	Wild-type	0.306	Negative	0%
28	IV	Wild-type	2.60	Negative	0%
39	IV	Wild-type	0.526	Negative	0%
44	IV	Wild-type	0.392	Negative	0%
46^b^	III	BRAF V600E	1.05	Negative	0%
54	IV	Wild-type	0.794	Negative	0%
55	IV	Wild-type	0.226	Negative	0%
61	IV	BRAF V600E	1.66	Positive	36.0%

^a^ Plasma samples obtained at baseline chemotherapy cycle unless noted; ^b^ Plasma sample obtained at 4^th^ chemotherapy cycle.
