# Supplementary material for: Multiplex detection of ctDNA mutations in plasma of colorectal cancer patients by PCR/SERS assay
Source: Nanotheranostics. 2020 Aug 25;4(4):224–32. doi: 10.7150/ntno.48905 (PMC7484630; doi:10.7150/ntno.48905)
Supplement: Supplementary file 1 — Supplementary figures and tables. [file ntnov04p0224s1.pdf]

Multiplex detection of ctDNA mutations in plasma of colorectal cancer patients by PCR/SERS assay

Nana Lyu[1], Vinoth Kumar Rajendran[1], Russell J. Diefenbach[2,3], Kellie Charles[4], Stephen J. Clarke[5], Alexander Engel[6], Sydney 1000 Colorectal Cancer Study Investigators[+], Helen Rizos[2,3], Mark P. Molloy[7,✉], and Yuling Wang[1,✉]

Supplementary Material

Sydney 1000 Colorectal Cancer Study Investigators[+]

Royal North Shore Hospital: Stephen Clarke, Alexander Engel, Anthony Gill, Andrew Kneebone, George Hruby, Nick Pavlakis, Connie Diakos, Justin Evans, Shahrir Kabir, Yasser Salama

The University of Sydney: Kellie Charles, Mark Molloy, Barbara Fazekas de St Groth, Margaret Allman-Farinelli, Adrian Bauman, Adrian Smith, Derek Hart, Haryana Dhillon, Madeleine King, Scott Byrne, Claudia Rutherford, Andrew Holmes, Nick King, Fatemeh Vafaeem Helen McGuire

Table S1. Sequences of oligonucleotides and the PCR amplicons used in this study.

**Oligonucleotides**	**5'-Sequence-3'**
BRAF V600E amplicon<br>(length: 100 bp)	ATAGGTGATTTTGGTCTAGCTACTGAGAAATCTCGATGGAGTGGGTCC<br>CATCAGTTTGAACAGTTGTCTGGATCCATTTTGTGGATGGTAAGAATT<br>GAGG
BRAF V600E F	CAGATCGTCATGTTC/iSpC3/ATAGGTGATTTTGGTCTAGCTACTGA
BRAF V600E R	/5Biosg/CCTCAATTCTTACCATCCACAAA
BRAF V600E probe nanotag	GAACATGACGATCTGTTTTT/3ThioMC6-D
KRAS G12V amplicon<br>(length: 92 bp)	CATTTTCATTATTTTTATTATAAGGCCTGCTGAAAATGACTGAATATAA<br>ACTTGTGGTAGTTGGAGCTGTTGGCGTAGGCAAGAGTGCCTTG
KRAS G12V F	/5Biosg/CATTTTCATTATTTTTATTATAAGGCCTG
KRAS G12V R	TCTGCACCAATGTAC/iSpC3/CAAGGCACTCTTGCCTACGCCAA
KRAS G12V probe nanotag	GTACATTGGTGCAGATTTTT/3ThioMC6-D
KRAS G13D amplicon<br>(length: 177 bp)	TGTGGTAGTTGGAGCTGGTGAGCGTAGGCAAGAGTGCCTTGACGATA<br>CAGCTAATTCAGAATCATTTTGTGGACGAATATGATCCAACAATAGAG<br>GTAAATCTTGTTTTAATATGCATATTACTGGTGCAGGACCATTCTTTGA<br>TACAGATAAAGGTTTCTCTGACCATTTTCATGA
KRAS G13D F	AGTCTGATGGCAGCA/iSpC3/TGTGGTAGTTGGAGCTGGTGAG
KRAS G13D R	/5Biosg/TCATGAAAATGGTCAGAGAAACC
KRAS G13D probe nanotag	TGCTGCCATCAGACTTTTTT/3ThioMC6-D

Modifications are as indicated. iSpC3: internal modification with C3 spacer, 5Biosg: 5'-biotin modified, 3ThioMC6-D: 3'-thiol modifier C6 S-S (disulfide). The text in red represents position of primers and the underlined text refers to the single nucleotide mutant points; The text in blue highlights complementary sequence in primers and probe nanotags.

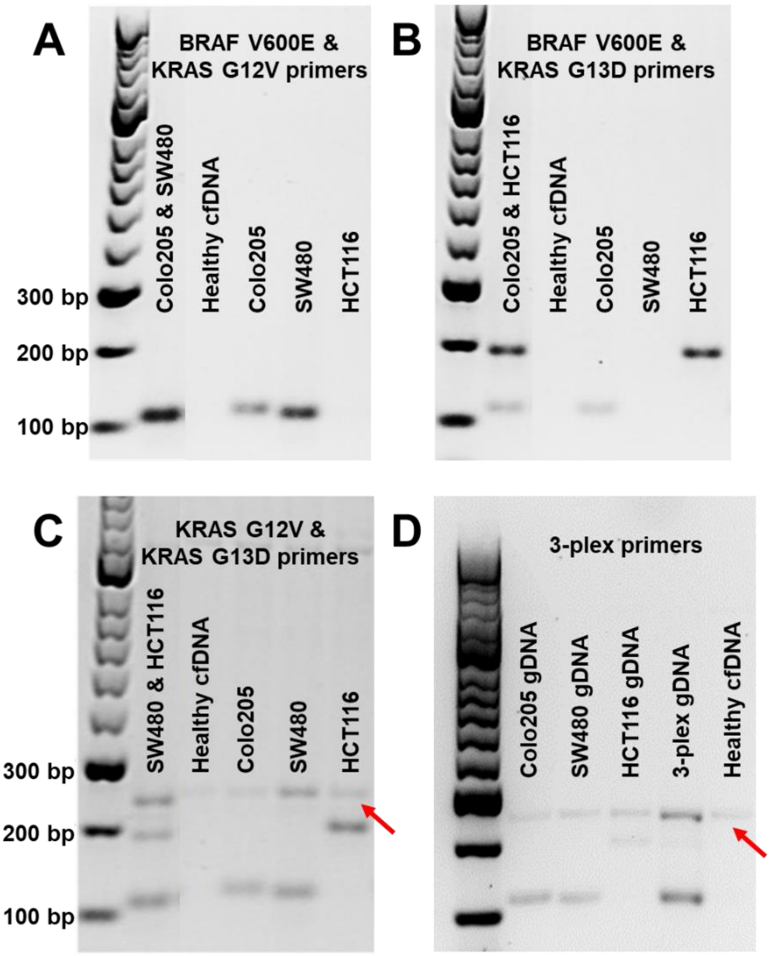

Figure S1. Specific multiplex amplification. The gel image of PCR products after amplification with multiplex primers. (A, B) Duplex amplification with BRAF V600E and KRAS G12V or KRAS G13D primers could accurately genotype the cell lines, demonstrating the specific amplification; (C, D) The duplex amplification with KRAS 12V and KRAS G13D primers, and 3-plex amplification showed additional bands, which may be contributed to the cross reaction of primers for the targets, where the KRAS G12V forward primer and KRAS G13D reverse primer amplified the template and obtained an additional amplicon with length of 229 bp. To avoid this additional amplicon being identified with SERS nanotags, the KRAS G12V forward primer and KRAS G13D reverse primer are both designed with a 5'-biotin modification, consequently, the additional amplicon had 5'-biotin on both ends and would not be identified by SERS nanotags.

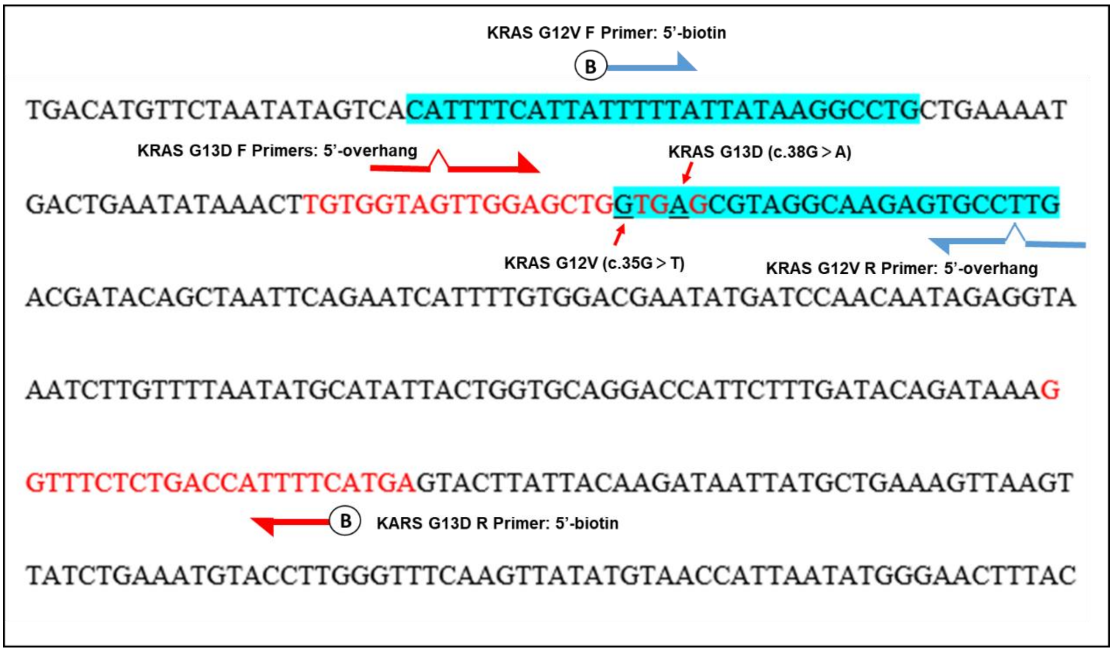

Figure S2. Cross reaction of primers for the targets. The KRAS G12V forward primer and KRAS G13D reverse primer could amplify the template and obtained an additional amplicon with length of 229 bp, the amplicon has 5'-biotin on both terminals but lack of 5'-overhang.

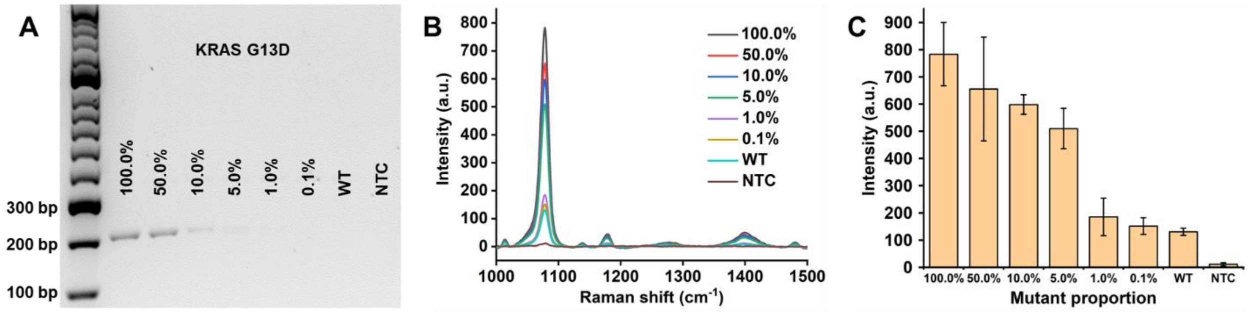

Figure S3. Detection of low levels of KRAS G13D mutation load. (A) Gel electrophoresis image, 5.0% of input targets could be recognized in the gel image (the ImageJ software was used for band quantification), (B) typical raw Raman spectra and (C) bar graph of average SERS intensities at 1076 cm^{-1} over a range of mutation loads for 10,000 input copies. NTC is the no template control. Error bar represents standard deviation (SD) of 3 independent experiments.

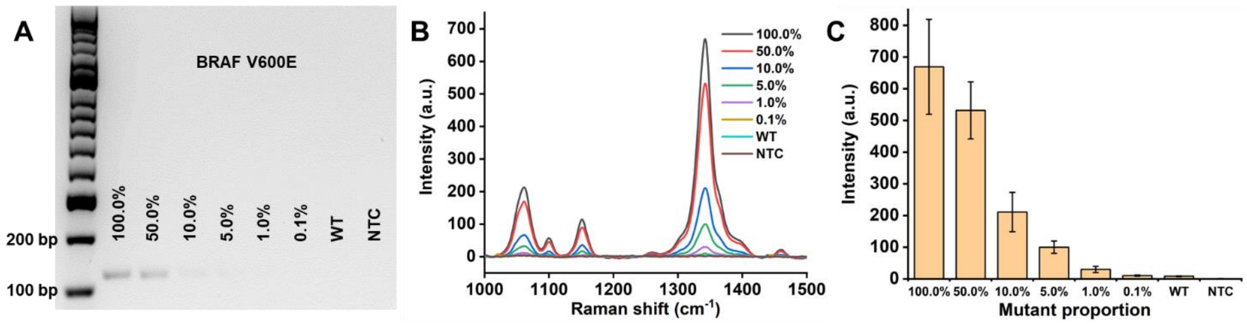

Figure S4. Detection of low levels of BRAF V600E mutation load. (A) Gel electrophoresis image, 5.0% of input targets could be recognized in the gel image (the ImageJ software was used for band quantification), (B) typical raw Raman spectra and (C) bar graph of average SERS intensities at 1342 cm^{-1} over a range of mutation loads for 10,000 input copies. NTC is the no template control. Error bar represents SD of 3 independent experiments.

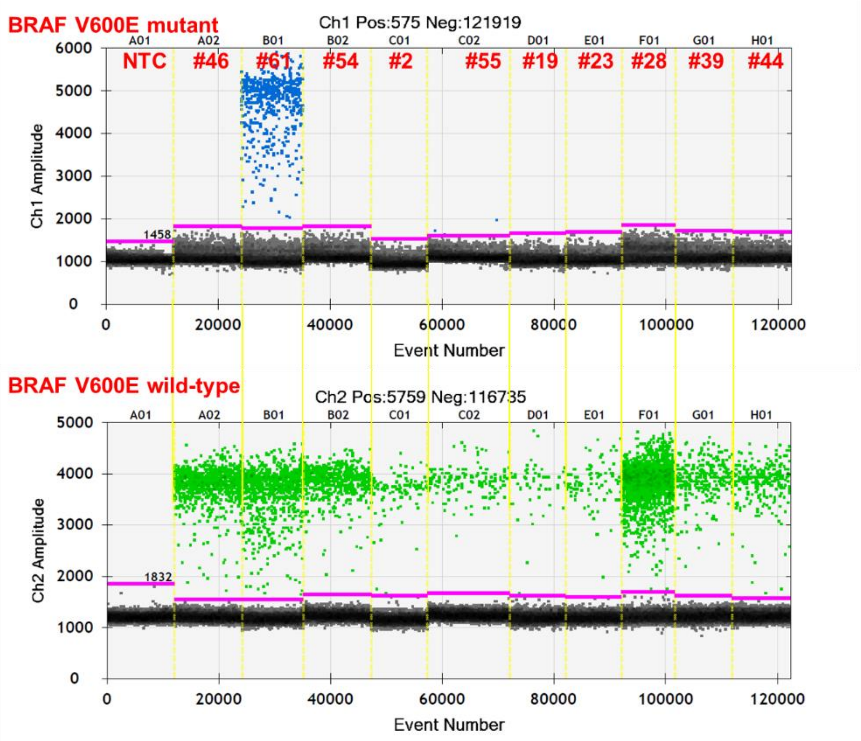

Figure S5. Detection of BRAF V600E mutation by ddPCR. The results show the BRAF V600E mutant allele frequency for #61 was high, other patients' samples were determined without BRAF V600E mutation, which are consistent with PCR/SERS assay.

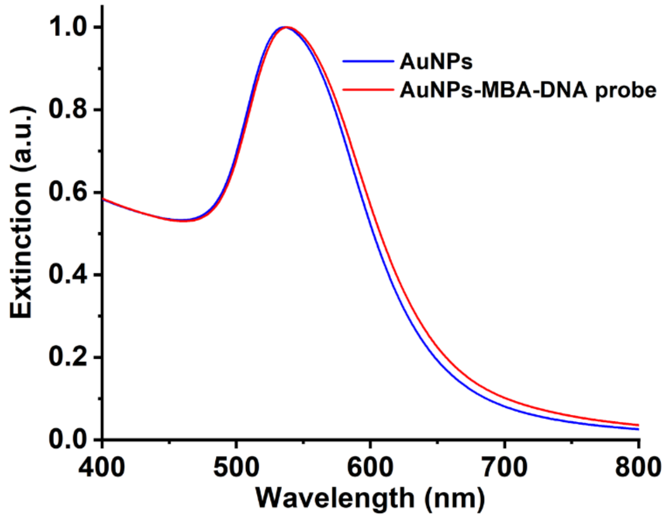

Figure S6. Characterization of SERS nanotags. UV-Vis spectrometry of AuNPs before and after coating with Raman molecule (MBA) and DNA probe (oligonucleotide).

Table S2. Reaction mixture for 3-plex PCR

Reaction mixture	Volume (µL)
5× Buffer A	5
BRAF V600E F (10 µM)	0.15
BRAF V600E R (10 µM)	0.15
KRAS G12V F (10 µM)	0.15
KRAS G12V R (10 µM)	0.15
KRAS G13D F (10 µM)	0.10
KRAS G13D R (10 µM)	0.10
dNTP mix (10 mM each)	0.4
$MgCl_2$ (25 mM)	1
BSA (5 µg/µL)	0.32
Ethylene glycol	2
Polymerase (5 U/µL)	0.08
gDNA or cfDNA	2
Water	8.4

Calculation

The SERS enhancement factor (EF) of gold nanoparticles (AuNPs) for Raman molecule TFMBA was determined as follows:

$$EF = \frac{I_{SERS}}{I_{Raman}} \times \frac{N_{Raman}}{N_{SERS}}$$

where I_{SERS} and I_{Raman} refer to Raman intensities of TFMBA at 1376 cm^{-1} with and without SERS substrate, respectively; and N_{SERS} and N_{Raman} are the number of TFMBA molecules excited in the laser spot during SERS and normal Raman measurements.

$\boldsymbol{I_{SERS}}$ was read as 13755 a.u., which is the absolute Raman intensity of TFMBA on AuNPs at 1376 cm^{-1} in the SERS spectrum.

$\boldsymbol{I_{Raman}}$ was read as 593 a.u., which is the absolute intensity of 0.2 M TFMBA in ethanol at 1396 cm^{-1} for normal Raman detection.

$\boldsymbol{N_{Raman}}$ was determined as 1.51×10^{15} molecules, which is calculated as follows:

The diameter (*d*) of laser spot through the liquid is 40 μm, the pathlength (*h*) is 10 mm. The volume of the laser path through can be calculated as:

$$V_{laser\ path} = \pi \left(\frac{d}{2}\right)^2 \times h = \pi \times (20\ \mu m)^2 \times 10\ mm = 1.257 \times 10^{-5}\ cm^3$$

Mole of 0.2 mol/L TFMBA in the laser spot path:

$$n_{TFMBA} = C_{TFMBA} \times V_{laser\ path}$$

$$= 0.2\ mol/L \times 1.257 \times 10^{-5}\ cm^3$$

$$= 2.514 \times 10^{-9} mol$$

N_{Raman} of 0.2 mol/L TFMBA in the laser spot path:

$$N_{Raman} = n_{TFMBA} \times Avogadro\ constant$$

$$= 2.514 \times 10^{-9} mol \times 6.022 \times 10^{23}$$

$$= 1.51 \times 10^{15}\ molecules$$

$\boldsymbol{N_{SERS}}$ is 6.38×10^{10} AuNPs molecules, which is calculated as follows:

The diameter (*d*) of AuNPs is 60 nm, the topological polar surface area of TFMBA (S_{TFMBA})

is 0.383 nm^2. The number of TFMBA molecules on each AuNP:

$$\boldsymbol{S_{AuNP}} = 4\pi r^2 = 4\pi\ (30\ nm)^2 = 11304\ nm^2$$

$$\boldsymbol{N_{TFMBA\ molecules\ on\ single\ AuNP}} = \frac{S_{AuNP}}{S_{TFMBA}} = \frac{11304\ nm^2}{0.383\ nm^2} = 29514.4\ TFMBA$$

We assume that 100 mL of 0.01% $AuCl_4{\cdot}3H_2O$ added was completely reduced to AuNPs.

$$\boldsymbol{Mole\ of\ Au^{3+}} = \frac{Weight\ of\ AuCl_4{\cdot}3H_2O}{Molecular\ weight\ of\ AuCl_4{\cdot}3H_2O} = \frac{0.01\ g}{393.8\ g/mol} = 2.54 \times 10^{-5}\ mol$$

$$\boldsymbol{Mass\ of\ Au^{3+}} = 2.54 \times 10^{-5} mol \times 196.97\ g/mol = 0.005\ g$$

Volume of a single AuNP

$$V = \frac{4\pi r^3}{3} = \frac{4\pi}{3}\ (30\ nm)^3 = 1.13 \times 10^{-16}\ cm^3$$

Mass of a single AuNP

$$m_{AuNP} = \rho_{Au} \times V_{AuNP} = 19.32\ g/cm^3 \times 1.13 \times 10^{-16}\ cm^3 = 2.18 \times 10^{-15}\ g$$

Total number of AuNPs:

$$\boldsymbol{N_{AuNPs}} = \frac{m_{Au^{3+}}}{m_{AuNP}} = \frac{0.005\ g}{2.18 \times 10^{-15}\ g} = 2.29 \times 10^{12}\ AuNPs$$

$$\boldsymbol{C_{AuNPs}} = \frac{N_{AuNPs}}{100\ mL} = \frac{2.29 \times 10^{12}\ AuNPs}{100\ mL} = 2.29 \times 10^{10}\ AuNPs/cm^3$$

The AuNPs used in this study were concentrated from 1.5 mL AuNPs to 0.2 mL, so the mole concentration should be:

$$\boldsymbol{C_{AuNPs\ after\ concentration}} = 2.29 \times 10^{10}/cm^3 \times \frac{1.5}{0.2} = 1.72 \times 10^{11} AuNPs/cm^3$$

$$\begin{aligned}\boldsymbol{N_{AuNPs\ in\ the\ laser\ path}} &= C_{AuNPs\ after\ concentration} \times V_{laser\ path} \\ &= 1.72 \times 10^{11}\ AuNPs/cm^3 \times 1.257 \times 10^{-5}\ cm^3 \\ &= 2.16 \times 10^6\ AuNPs\end{aligned}$$

$$\begin{aligned}\boldsymbol{N_{SERS\ (TFMBA\ on\ AuNPs)}} &= N_{AuNPs\ in\ the\ laser\ path} \times N_{TFMBA\ molecules\ on\ single\ AuNP} \\ &= 2.16 \times 10^6\ AuNPs\ \times 29514.4\ TFMBA \\ &= 6.38 \times 10^{10}\ TFMBA\ molecules\end{aligned}$$

$$\boldsymbol{EF} = \frac{I_{SERS}}{I_{Raman}} \times \frac{N_{Raman}}{N_{SERS}} = \frac{13755}{593} \times \frac{1.51 \times 10^{15}}{6.38 \times 10^{10}} = 5.49 \times 10^6$$
